# Effect of Impurities Control on the Crystallization and Densification of Polymer-Derived SiC Fibers

**DOI:** 10.3390/nano11112933

**Published:** 2021-11-02

**Authors:** Young-jun Joo, Sang-hyun Joo, Hyuk-jun Lee, Young-jin Shim, Dong-geun Shin, Kwang-youn Cho

**Affiliations:** 1 Fibrous Ceramics & Aerospace Materials Center, Korea Institute of Ceramic Engineering and Technology, Jinju 52858, Korea; kicet_joo@kicet.re.kr (Y.-j.J.); judy@kicet.re.kr (S.-h.J.); kyg325@naver.com (H.-j.L.); sksms182@naver.com (Y.-j.S.); dgshin73@kicet.re.kr (D.-g.S.); 2 Division of Nano & Advanced Materials Engineering, Gyeongsang National University, Jinju 52858, Korea

**Keywords:** polycarbonsilane, SiC fiber, impurities control, crystallization, densification

## Abstract

The polymer-derived SiC fibers are mainly used as reinforcing materials for ceramic matrix composites (CMCs) because of their excellent mechanical properties at high temperature. However, decomposition reactions such as release of SiO and CO gases and the formation of pores proceed above 1400 °C because of impurities introduced during the curing process. In this study, polycrystalline SiC fibers were fabricated by applying iodine-curing method and using controlled pyrolysis conditions to investigate crystallization and densification behavior. Oxygen and iodine impurities in amorphous SiC fibers were reduced without pores by diffusion and release to the fiber surface depending on the pyrolysis time. In addition, the reduction of the impurity content had a positive effect on the densification and crystallization of polymer-derived SiC fibers without a sintering aid above the sintering temperature. Consequently, dense Si-Al-C-O polycrystalline fibers containing β-SiC crystal grains of 50~100 nm were easily fabricated through the blending method and controlled pyrolysis conditions.

## 1. Introduction

Silicon carbide (SiC) fiber with excellent oxidation resistance, high tensile strength, elastic modulus at high temperature is mainly used as a reinforcing material for ceramic matrix composites (CMCs) [1,2,3]. SiC fibers are generally manufactured by the processes of melt-spinning, curing, and pyrolysis using polycarbosilane (PCS) as a ceramic precursor [4,5]. Especially, the polymer-derived SiC fibers can be largely divided into amorphous SiC fibers and polycrystalline SiC fibers depending on the oxidation resistance temperature, and various manufacturing methods have been studied to fabricate high-performance polycrystalline SiC fibers [6,7].

The curing methods essential for conversion from polymer fiber to ceramic fiber are classified into oxidation curing method [8,9,10], electron beam (EB) curing method [11,12,13], and chemical vapor curing (CVC) method [14,15,16], respectively. The oxidation curing method, which forms cross-linking such as Si-O-Si and Si-O-C bonds between PCS structures by reaction of PCS and hot air, is an inexpensive and easy method. However, oxygen as a cross-linker reduces the heat resistance of SiC fibers and causes problems in fabricating dense and polycrystalline SiC fibers.

The EB curing method was developed to manufacture high-heat resistant SiC fibers through the curing process without oxygen. The EB-cured PCS fibers are relatively easily converted into polycrystalline SiC fibers above 1500 °C through the formation of radical bonds such as Si-Si and Si-C under a strong electron beam. However, the EB curing method is expensive and difficult to access in various industries because the EB irradiation equipment is very sophisticated and high power.

Among CVC methods, the iodine curing method induces the oxidation reaction of PCS at 120 °C or less, so it has the advantage of being applicable to low-melting point or low-molecular weight PCS. However, the amorphous SiC fiber fabricated by the iodine curing method contains iodine impurity as a cross-linking accelerator and oxygen impurity as a cross-linking agent. As a result, this amorphous SiC fiber not only has lower high-temperature heat resistance but is also difficult to convert to polycrystalline SiC fibers.

As mentioned above, it is known that amorphous SiC fibers fabricated by oxidation curing and CVC methods contain oxygen impurity in the vicinity of 10% [10,17]. In amorphous SiC fibers, oxygen impurities exist in oxygen compounds such as the SiO_x_C_y_ and SiO_2_ phase and are decomposed into SiO and CO gases at high temperatures (≥1300 °C) [18,19]. Consequently, it means that oxygen introduced as a cross-linking agent reduces the heat resistance of amorphous SiC fibers and prohibits conversion to polycrystalline SiC fibers with high-heat resistance. In this paper, iodine-cured PCS fibers were converted into amorphous SiC fibers via applying a controlled pyrolysis process, and then the crystallization behavior was investigated using amorphous SiC fiber with controlled oxygen content.

PCS containing various organometallic compounds have been synthesized to fabricate polycrystalline SiC fibers by inhibiting the decomposition of oxygen impurities during the conversion from amorphous SiC to crystalline SiC [20,21]. In particular, Si-Al-C-O fibers manufactured using polyaluminocarbosilane (PACS) are representative ceramic grade fibers having a strength retention temperature of 1700 °C [21,22]. These modified PCS are usually synthesized using an autoclave or reflux system, but it is known that the blend method is good for mass production and easy application.

Therefore, in this work, amorphous SiC fibers without sintering aid were fabricated using the iodine curing method and controlled pyrolysis conditions, and then pyrolyzed at 1600 and 1800 °C to investigate the crystallization behavior of the polymer-derived SiC fibers. As a result, the amorphous SiC fibers prepared through the novel process of impurities control were converted into dense SiC polycrystalline fiber without additives such as sintering aids. In addition, Al-added PCS was easily prepared by the solution blend method, and dense Si-Al-C-O polycrystalline fibers were successfully fabricated by applying the crystallization behavior of SiC fibers investigated in this study.

## 2. Experimental Procedure

### 2.1. Raw Materials

Polycarbosilane (PCS) having weight average molecular weight (M_w_) of 3327, number average molecular weight (M_n_) of 1565, and melting point of 185 °C, respectively, was purchased from ToBeMTech Co., Ltd. (Yongin-si, Gyeonggi-do, Korea). Iodine (extra pure 99.0%) as a cross-linking accelerator was purchased from Samchun pure chemical Co., Ltd. (Gangnam-gu, Seoul, Korea). Aluminum acetylacetonate (anhydrous 99.0%) and toluene (anhydrous 99.7%) were purchased from Sigma-Aldrich Inc. (Burlington, MA, USA).

### 2.2. Preparation of Polycrystalline SiC Fibers

**Figure 1:**
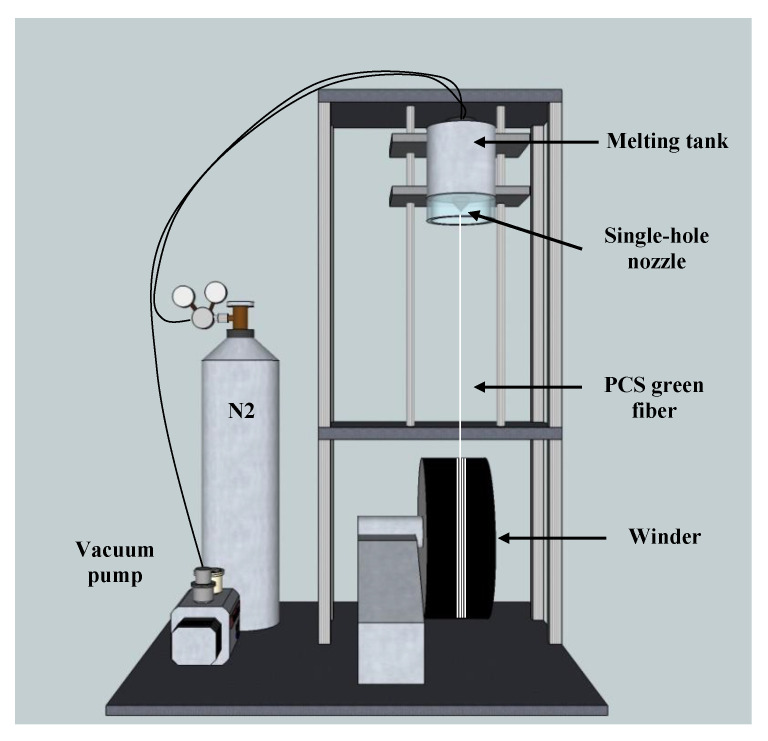
The schematic drawing of melt-spinning machine equipped with a single-hole nozzle.

PCS was melted at 180–190 °C for 3 h in N_2_ atmosphere using single-hole spinning machine (DAEHO I&T Co., Ltd., Jinju-si, Gyeongsangnam-do, Korea), as shown in Figure 1. Subsequently, PCS green fibers with a diameter of about 20 μm were prepared through a winder speed of 1000 rpm. The chemical vapor curing method using iodine was adopted to fabricate the infusible PCS fiber because of the relatively low-melting point of PCS. The PCS green fiber and iodine in a weight ratio of 1:1 were placed together in a graphite mold, and then heat-treated up to 180 °C at a heating rate of 10 °C/min in low vacuum. PCS green fibers were converted into cured PCS fibers with a bright yellow by reaction with iodine. To control the content of oxygen impurities during the pyrolysis process, the iodine-cured PCS fibers were heat-treated at 1400 °C for 2, 4, and 6 h in an inert atmosphere. Finally, the amorphous SiC fibers were heat-treated at 1800 °C in an inert atmosphere to analyze the crystallization behavior according to the oxygen impurity content.

### 2.3. Preparation of Polycrystalline Si-Al-C-O Fibers

The PCS solution was prepared using toluene, and aluminum acetylacetonate in an amount of 1, 3, and 5 wt% based on the weight of the PCS was added and stirred at room temperature for 6–12 h. The Al-added PCS solutions were vacuum-dried in the range of 150–180 °C. During the melt-spinning process, the unreacted Al source causes the problem in reducing the diameter of the fiber and making it continuous. Therefore, the Al-added PCS bulk was again dissolved using toluene, and then filtered using a filter paper with a 0.08 μm particle. The Al-added PCS solution prepared by removing the unreacted Al source was vacuum-dried under the same conditions, and the modified PCSs were named PCS-Al1, PCS-Al3, and PCS-Al5, respectively. The amorphous and polycrystalline SiC fibers containing Al content fabricated using PCS-Al precursor in the same method as described above.

### 2.4. Measurements

Fourier-transform infrared spectroscopy (FT-IR, L1860116, PerkinElmer, Waltham, MA, USA) analysis was performed in the range of 450–4000 cm^−1^. The PCS and modified PCS were pulverized into powder and measured in attenuated total reflection (ATR) mode by 16-times scan.

Thermogravimetric analysis (TG, STA449 F3, NETZSCH, Gebrüder-Netzsch-Straße 19, 95100 Selb, Germany) was conducted at the heating rate of 10 °C/min up to 1600 °C to confirm the decomposition behavior and ceramic yield. PCS of 10.0 mg was placed in an alumina crucible and analyzed after stabilization for 30 min.

The morphology and element distribution of polymer-derived SiC fibers were observed by field emission-scanning electron microscopy (FE-SEM, JSM-7610F, JEOL, Tokyo, Japan). Pt coating was performed using an ion coater for 70 s. Energy dispersive spectroscopy (EDS) was measured by repeating 5–10 times after pulverizing polymer-derived SiC fibers into fine powder. The standard deviation for the element content measurements of Si, C, O, and I were 4.81%, 3.95%, 2.06%, and 0.01%, respectively.

X-ray diffraction analysis (XRD, DMAX 2500, Rigaku, Akishima-shi, Tokyo, Japan) in the range of 20–80° was conducted to analyze the phase and crystallinity of the amorphous and polycrystalline SiC fibers. The measurement was carried out by scanning at 8° per minute in continuous mode.

The microstructural analysis of SiC-polycrystalline fibers was carried out by transmission electron microscopy (TEM, FEI Titan Themis Z, Thermo Fisher Scientific, Waltham, MA, USA). TEM analysis samples were prepared using focused ion beam (FIB, Helios G4 UC, Thermo Fisher Scientific, Thermo Fisher Scientific, Waltham, MA, USA).

## 3. Results and Discussion

### The Crystallization Behavior of Polymer-Derived SiC Fibers

**Figure 2:**
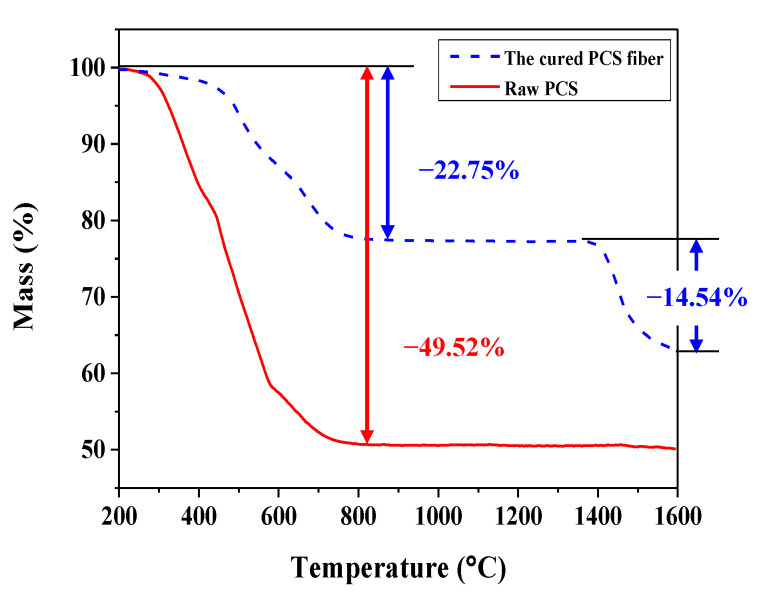
TG curves of raw PCS and iodine-cured PCS fibers.

Figure 2 shows the decomposition behavior of raw PCS and iodine-cured PCS fiber up to 1600 °C. The weight loss of raw PCS started at about 250 °C and ended at about 800 °C. On the other hand, the weight loss of iodine-cured PCS fibers started at about 400 °C as a result of the condensation and dehydrogenation reaction that occurred in the curing process. In addition, the ceramic yield of iodine-cured PCS fiber at 1000 °C was increased by about 25% compared that of raw PCS. However, the weight loss of about 14.54% was additionally formed in the range of 1400–1600 °C due to iodine and oxygen impurities introduced during the curing process. The amorphous SiC fibers were prepared by heat treatment in an inert atmosphere for 2, 4, and 6 h to control the decomposition occurring at 1400–1600 °C.

**Figure 3:**
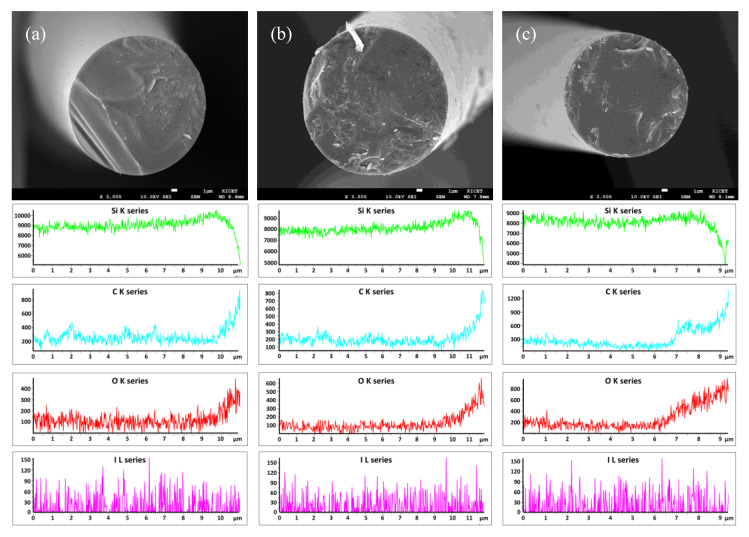
SEM-EDS images of the polymer-derived SiC fibers fabricated at 1400 °C for (**a**) 2, (**b**) 4, and (**c**) 6 h.

Figure 3 shows the SEM-EDS results of amorphous SiC fibers depending on the heat treatment time. The polymer-derived SiC fibers fabricated at different times showed smooth cross-sectional surface without pores. However, the distribution of element content was changed according to the heat treatment time. SiC fibers fabricated through iodine curing method showed a lot of carbon and oxygen distribution on the surface. As the heat treatment time increased, the intensity of carbon and oxygen on the surface increased as the heat treatment time increased, but the distribution of silicon and iodine was maintained.

Table 1 exhibits the tendency of the element content in the amorphous SiC fiber prepared at different times. EDS mapping was performed on the pulverized powder to confirm the overall tendency. As a result, the iodine content introduced during the curing process was hardly observed in all samples, and the oxygen content decreased continuously with increasing pyrolysis time. These results indicated that the pyrolysis process at 1400 °C for a long-time has an effect on removal of impurities from the amorphous SiC fiber without pores or defects.

**Figure 4:**
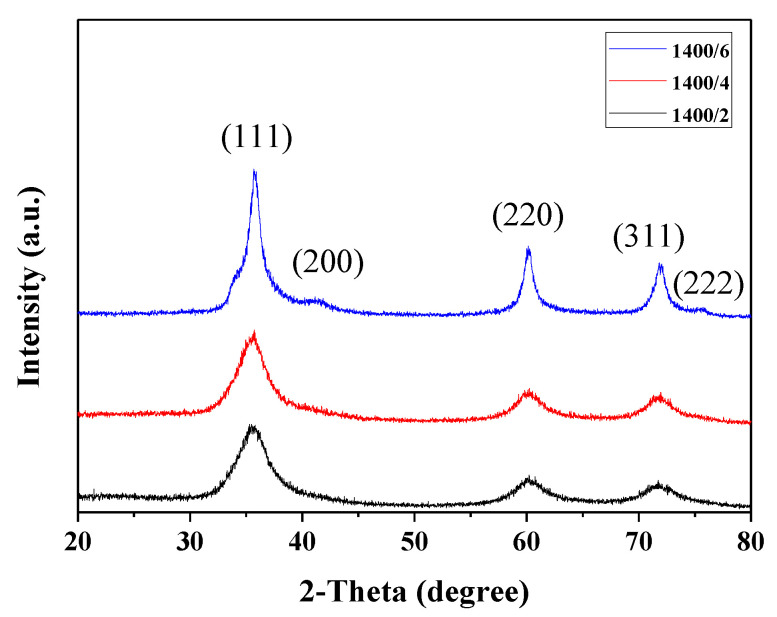
X-ray diffraction patterns of the polymer-derived SiC fiber with different heat treatment times.

Figure 4 shows the X-ray diffraction patterns of the polymer-derived SiC fibers. The three main peaks at 36°, 41°, 60°, 70°, and 76° correspond to the (111), (200), (220), (311), and (222) planes of the β-SiC crystal. The polymer-derived SiC fibers with different pyrolysis times showed broad peaks overall. In particular, it was observed that SiC fibers prepared at 1400 °C for 6 h exhibited a narrower full width at half maximum (FWHM) and additional crystal plane compared to fibers prepared at 2 and 4 h due to crystallization by decomposition of the SiO_x_C_y_ phase.

**Figure 5:**
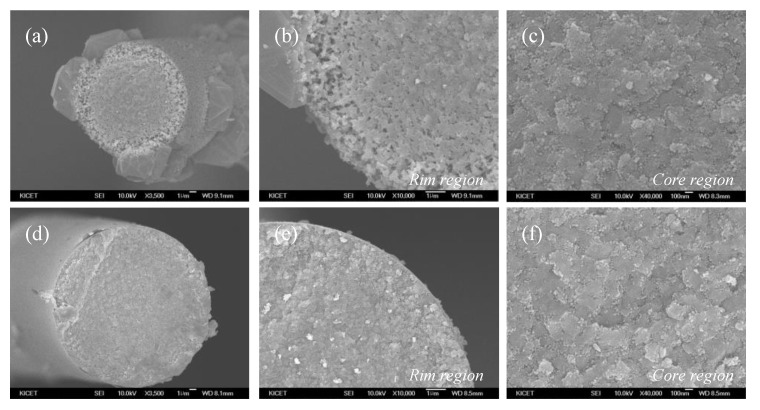
SEM images of polymer-derived SiC fibers fabricated at 1800 °C with (**a**–**c**) uncontrolled and (**d**–**f**) controlled pyrolysis process.

Figure 5a–c shows SEM images of polymer-derived SiC fibers fabricated at 1800 °C without impurity control process. Additionally, Figure 5d–f shows the dense polymer-derived SiC fiber fabricated by heat treatment at 1800 °C again after controlling the impurity contents at 1400 °C for 6 h. The polymer-derived SiC fiber in Figure 5a,b had a porous layer of about 4.9 μm and large SiC crystals on the surface compared with that of Figure 5d,e. On the other hand, the core region of the SiC fiber had a very dense surface and similar grain size as shown in Figure 5c,f. These results showed that impurity control had a great effect on the uniform densification of SiC fibers and suppression of the formation of the large SiC crystals on the surface.

**Figure 6:**
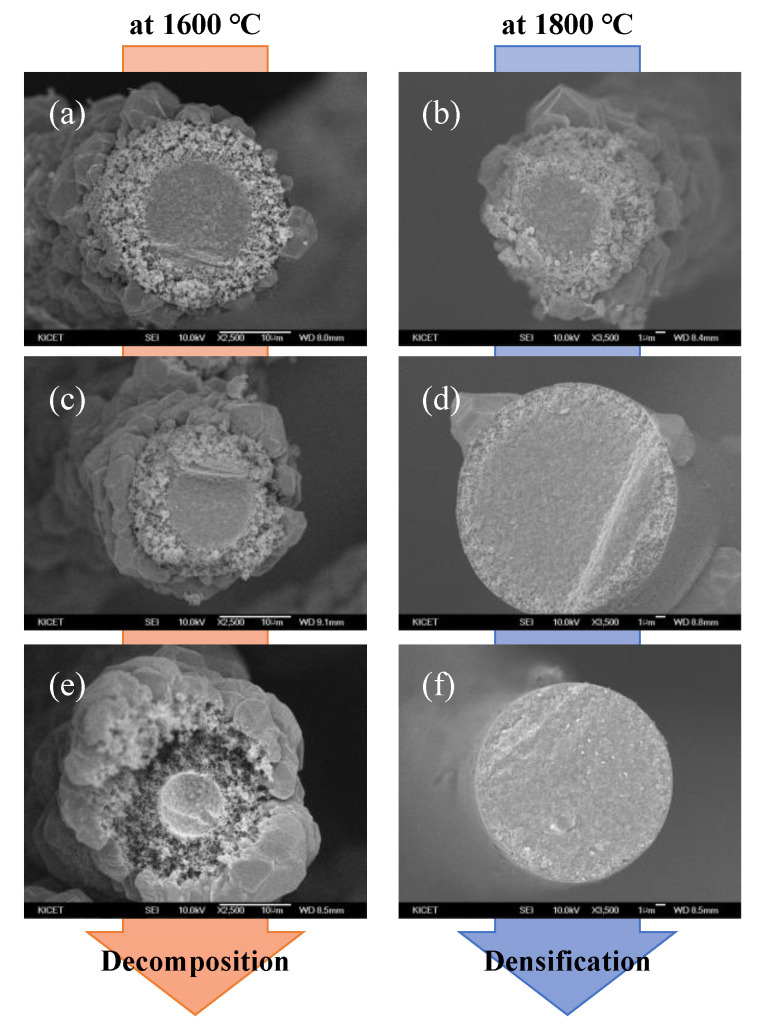
The cross-sectional SEM images of polycrystalline SiC fibers fabricated at 1600 and 1800 °C using fibers with controlled impurity content at 1400 °C for (**a**,**b**) 2, (**c**,**d**) 4, and (**e**,**f**) 6 h.

Figure 6 shows the cross-sectional SEM images of the polycrystalline SiC fibers fabricated using the amorphous SiC fiber with controlled impurity content. The amorphous SiC fibers prepared by pyrolysis for 2, 4, and 6 h were additionally heat treatment at 1600 and 1800 °C in an inert atmosphere to confirm the crystallization behavior. In the polycrystalline SiC fiber heat-treated at 1600 °C, coarsening of crystal grains was observed on the fiber surface despite the use of amorphous SiC fiber with controlled oxygen content. On the other hand, the polycrystalline SiC fiber fabricated at 1800 °C showed a dense surface due to the control effect of the impurity content in the fabrication stage of amorphous SiC fibers.

As shown in Figure 3 and Table 1, the amorphous SiC fiber fabricated through iodine curing method showed decomposition by the release of residual iodine with SiO and CO gases above 1400 °C without pores. The surface of this fiber contains high oxygen and carbon contents compared to the inside. In fact, impurity gases that decompose in the vicinity of the surface can be easily released and removed during heat treatment, but the impurity gases generated in the core region diffused out to the surface, leaving large pores between the surface and the core [22,23].

**Figure 7:**
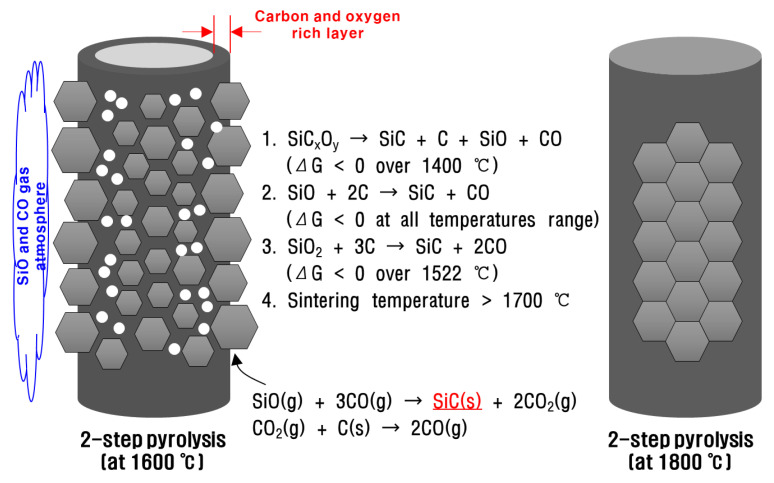
The schematic drawing of the crystallization behavior of polymer-derived SiC fibers.

For this reason, the polymer-derived SiC fibers fabricated below sintering temperature (at 1600 °C) showed dense core region and porous rim region despite the control of impurity contents due to the lower sintering temperature and residual impurities. On the other hand, above the sintering temperature (at 1800 °C), the polymer-derived SiC fiber not only induced SiC crystal growth by reacting SiO gas, SiO_2_, and free carbon generated in the core region, but also filled the micropores formed in the decomposition temperature region by sintering as shown in Figure 6f. The crystallization and degradation behaviors of polymer-derived SiC fibers with controlled impurity content are summarized using SEM-EDS results in Figure 7. In other words, long-time heat treatment in the stage of amorphous SiC fiber means that it is possible to reduce the content of impurities and help in the manufacturing of polycrystalline SiC fibers without sintering aid when oxygen content is introduced by the curing process.

**Figure 8:**
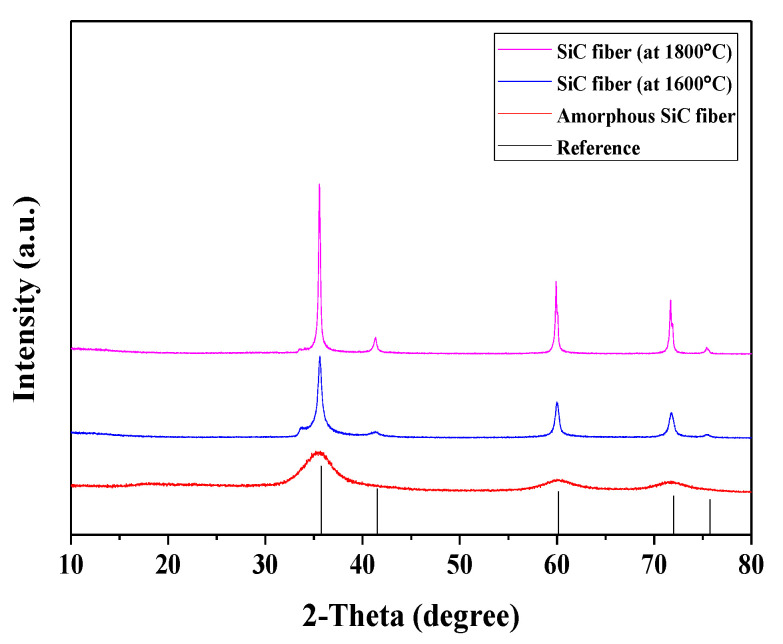
The schematic drawing of the crystallization behavior of polymer-derived SiC fibers.

Figure 8 shows the X-ray diffraction patterns of amorphous SiC fibers and polycrystalline SiC fibers fabricated via the control of impurity contents. The polycrystalline SiC fibers fabricated at 1600 and 1800 °C showed sharp peaks at 36°, 41°, 60°, 70°, and 76° compared to those of amorphous SiC fibers.

**Figure 9:**
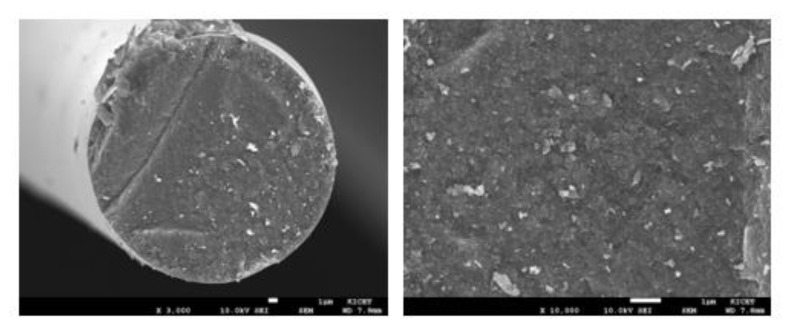
The cross-sectional SEM images of the polycrystalline Si-Al-C-O fibers fabricated via the control of impurity contents.

PCS with aluminum source were prepared through the blend method, which is a method for easily reacting PCS with organometallic compounds. Al-added PCS fibers were heat-treated with iodine at a weight ratio of 1:1 in the same manner as described above. Then, polycrystalline Si-Al-C-O fibers were fabricated by pyrolyzing at 1400 and 1800 °C applying an impurity control process. Figure 9 shows a cross-sectional SEM image of polycrystalline Si-Al-C-O fibers. It was confirmed that the polycrystalline Si-Al-C-O fibers prepared through iodine curing had a very dense and clean surface due to the influence of Al as a sintering aid as well as a controlled heat treatment process.

**Figure 10:**
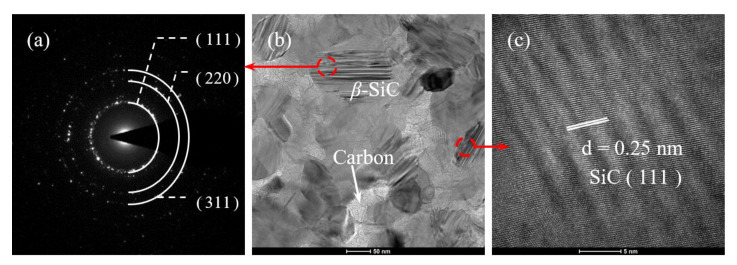
(**a**) SAD pattern and (**b**,**c**) TEM images of polycrystalline Si-Al-C-O fiber fabricated using the controlled pyrolysis condition.

Figure 10 shows selected area diffraction (SAD) pattern and TEM images of polycrystalline Si-Al-C-O fiber fabricated at 1800 °C using controlled pyrolysis conditions. In Figure 10a, the SAD pattern showed a ring pattern similar to that of an amorphous SiC fiber because fine SiC crystal grains and free carbon were alternately stacked as shown in Figure 10b. In addition, Figure 10b,c shows polycrystalline SiC grains with a diameter of 50–100 nm.

## 4. Conclusions

In this work, the crystallization behavior of polymer-derived SiC fibers was investigated by applying controlled pyrolysis conditions without sintering aid. It was confirmed that oxygen and iodine impurities gradually decreased without defects by long-time pyrolysis conditions of the fiber to which iodine curing was applied. Additionally, SiC fibers fabricated at 1800 °C exhibited the release of SiO and CO gases to the surface and densification behavior from the inside during crystal growth of β-SiC. On the other hand, the polycrystalline SiC fiber fabricated at 1600 °C was not densified despite the use of amorphous SiC fibers with low-oxygen content. This meant that a specific temperature above the sintering temperature was required as well as a reduction in the content of oxygen impurities for dense SiC-polycrystalline fibers. As a result, the polycrystalline Si-Al-C-O fibers prepared using controlled pyrolysis conditions had a dense structure including fine crystal grains of 50–100 nm.

## Figures and Tables

**Table 1:** EDS mapping of amorphous SiC fiber depending on the heat treatment time.

Elmt	1400/2		1400/4		1400/6	
	wt%	at%	wt%	at%	wt%	at%
Si	45.62	27.39	44.96	26.64	48.03	29.14
C	45.27	63.20	48.52	66.69	46.04	64.68
O	9.02	9.39	6.42	6.66	5.88	6.18
I	0.09	0.01	0.01	0.01	0.04	0.01
C/Si ratio	-	2.31	-	2.50	-	2.21
